# Overexpression of the Ubiquitin Ligase RNF182 Is Associated with High-Grade Gliomas

**DOI:** 10.3390/cancers18081217

**Published:** 2026-04-11

**Authors:** Veronica Russo, Miriam Russo, Maria Antonietta Oliva, Marika Alborghetti, Matteo Caridi, Felice Giangaspero, Antonietta Arcella

**Affiliations:** 1 IRCCS Neuromed, 86077 Pozzilli, Italy; veronica.2306@hotmail.it (V.R.); miriamrusso39@gmail.com (M.R.); mariaantonietta.oliva@neuromed.it (M.A.O.); marika.alborghetti@uniroma1.it (M.A.); neuropatologia@neuromed.it (F.G.); 2 Department of Neuroscience, Mental Health and Sensory Organs, Sapienza University of Rome, 00189 Rome, Italy; 3 Department of Physiology and Pharmacology, Sapienza University, 00185 Rome, Italy; matteo.caridi@uniroma1.it

**Keywords:** glioblastoma, RNF182, UPS, cell growth, silencing

## Abstract

**Background**: Glioblastoma (GBM) is the most common and aggressive brain tumor in adults. Changes in the ubiquitination system in GBM cells can promote uncontrolled tumor growth and reduce the effectiveness of treatments. However, the exact targets and regulatory elements of the ubiquitin–proteasome system involved in GBM are still not well understood. **Methods**: All data were obtained by using in silico analysis, immunohistochemistry, Western blot, RT-qPCR, gene silencing and proliferation assay. **Results**: Computational and protein analyses show that aggressive gliomas have higher expression of the RING ligase RNF182, with significantly greater levels in glioblastoma (GBM) than in low-grade gliomas. Elevated RNF182 is strongly associated with GBM growth. Experiments using siRNA to inhibit RNF182 in the human glioblastoma cell line U87MG significantly reduced cell proliferation, suggesting that RNF182 promotes tumor growth and may be a potential therapeutic target. **Conclusions**: These findings create a connection between the ubiquitin–proteasome system and the unchecked growth observed in GBM, identifying RNF182 as a new marker associated with GBM proliferation and an additional target for GBM treatment.

## Simple Summary

Glioblastoma (GBM) is the most common and aggressive brain tumor affecting adults. Dysregulation of the ubiquitination system has been shown to reduce treatment efficacy and contribute to uncontrolled tumor growth in various cancers, particularly in GBM. However, the specific targets and regulatory components of the ubiquitin–proteasome system involved in GBM remain largely unexplored. Our analyses show that aggressive gliomas exhibit increased expression of the RING ligase RNF182, with significantly higher levels in GBM than in low-grade gliomas. Silencing of rnf182 in the GBM cell line U87MG reduces cell proliferation, suggesting that the protein promotes tumor growth. Overall, RNF182 emerges as a potential biomarker and therapeutic target, linking the ubiquitin–proteasome system to GBM progression.

## 1. Introduction

Glioblastoma (GBM), which accounts for 60% of all gliomas, is the most common primary intracranial tumor. Also referred to as primary or de novo GBM, it makes up around 90% of GBM cases and usually impacts individuals who are 62 years old and above [1,2]. These tumors can be further classified according to the mutation of the IDH1 gene; generally, IDH-mutated GBM represent secondary GBM (about 10% of cases), which gradually progresses from low-grade astrocytoma and often occurs in patients aged 40–50 years [3,4,5]. In a recent study, the molecular profile of glioma shows a new classification: IDH1-mutated glioma is clinically associated with a long survival and a good prognosis, and aggressiveness and poor survival have instead been associated with the TERT promoter mutation, which is mainly represented in primary GBM [6,7]. A poor prognosis has also been linked to the deletion of CDKN2A-p16 [8,9]. Additionally, five molecular subclasses—mesenchymal, classical (or proliferative), proneural, neural, and G-CIMP—have been identified by genomic investigations [10,11]. Both primary and secondary glioblastomas have a mean lifespan of 12 to 15 months and a high rate of tumor recurrence, indicating that there is no discernible difference in patient survival between them despite advancements in our understanding and molecular characterization of glioblastomas [1,12]. The standard treatment for patients with GBM involves surgical resection, complemented by radiotherapy and adjuvant chemotherapy using the alkylating agent temozolomide (TMZ) [13]. While certain molecular characteristics have been suggested as predictive biomarkers for treatment response to alkylating agents, such as the methylation status of the O6-methylguanine-DNA-methyltransferase (MGMT) promoter, their clinical applicability remains limited [14,15]. Several factors have been proposed to explain the gliomas resistance, including the diffuse infiltrative nature of GBM, which complicates complete resection; the significant heterogeneity of GBM, which encompasses various molecular pathways; and, more recently, the identification of stem cell-like tumorigenic traits, such as promoting angiogenesis, uncontrolled cell proliferation, resistance to cell death, and genomic instability and mutation [16,17]. Although efforts have been made to identify molecular pathways and potential therapeutic targets involved in gliomagenesis, total tumor resection followed by adjuvant chemotherapy and radiotherapy remains the standard of care [13,18,19]. However, in most cases, therapy is ineffective in controlling the tumor growth, which often recurs after a period of time that varies from patient to patient. Furthermore, the intratumoral heterogeneity of GBM complicates the clinical outcome of the therapy, demanding the discovery of novel therapeutic targets for this disease [20,21]. The ubiquitin–proteasome system (UPS) emerged as an important component of the cellular machinery that controls protein fate and activity. By modulating the ubiquitylation state of a target protein, the UPS regulates key biological functions, including growth, metabolism, differentiation and development [22,23]; therefore, it represents a promising target for developing new therapeutic strategies for GBM. RNF182 promotes the ubiquitination and proteasomal degradation of ATP6V0C [24]. Yeast two-hybrid screening, overexpression, and co-immunoprecipitation have demonstrated an interaction between RNF182 and ATP6V0C both in vitro and in vivo. Additionally, RNF182 contributes to the suppression of TLR-mediated innate immune responses by inducing Lys48-linked ubiquitination and degradation of the NF-κB subunit RELA [25]. RNF182 is a low-abundance cytoplasmic protein expressed preferentially in the brain. Its expression was elevated in post-mortem Alzheimer’s disease (AD) brain tissue [24]. RNF182 protein possessed an E3 ubiquitin ligase activity and stimulated the E2-dependent polyubiquitination in vitro. The role of this protein has not previously been investigated in GBM. In this study, for the first time we propose to analyze the expression of RNF182 in GBM. The aim of our investigation is to elucidate the role of RNF182 and its involvement in gliomagenesis, with the final goal of identifying a novel biomarker for glioblastoma.

## 2. Materials and Methods

### 2.1. Computational Analysis

We analyzed two public RNA-seq datasets: TCGA and CPTAC. TCGA and CPTAC contain 166 and 99 primary GBM samples, respectively. Several clinical variables, including overall survival, were available and obtained from both cBioPortal (https://www.cbioportal.org/datasets, accessed on 21 March 2025) and Gliovis (https://gliovis.bioinfo.cnio.es/, accessed on 21 March 2025).

Data were analyzed with RStudio (RStudio: Integrated Development for R. RStudio, PBC, Boston, MA, USA) using the following packages: “survival,” “survminer,” “ggplot2,” “readxl,” “RCircos,” “WGCNA,” “clusterProfiler,” “org.Hs.eg.db,” “AnnotationDbi,” and “KEGGREST.” For all analyses, the latest available versions of the respective packages as of April 2025 were used.

The RNF183 family was presented using the R package RCircos. Global expression profiles were standardized using a base-2 logarithmic transformation (log_2_). Standard statistical analyses and density plots were generated for each dataset to assess RNF182 gene expression levels.

The Shapiro–Wilk test was employed to assess the normality of RNF182 expression. Considering the non-normal distribution (*p* < 0.0001), differences in gene expression among groups were analyzed using the Mann–Whitney test and the Kruskal–Wallis test. Statistical analyses were performed using RStudio (Version 2023.06.02+561) and Microsoft Excel (Version 16.16.3).

### 2.2. Cell Culture and Human Tissue Biopsies

The continuous human GBM cell line (U87MG) was purchased from Sigma Aldrich Collection (LGCPromochem, Teddington, UK). The cells were cultured in DMEM (Dulbecco’s Modified Eagle’s Medium) supplemented with 10% FBS (Fetal Bovine Serum), 2 mmol/L-glutamine, 100 IU/mL of Penicillin, and 100 g/mL of Streptomycin, at 37 °C, 5% CO_2_, and 95% humidity. The human tissue biopsies were obtained from Neuromed patients who had their gliomas and GBM surgically removed. They granted their informed consent to proceed with scientific research.

### 2.3. Immunohistochemistry

RNF182 protein expression was analyzed by immunohistochemistry in tissues from 18 patients with low-grade glioma and 18 patients with GBM. Paraffin-embedded tissue sections, 4 micrometers thick, were deparaffinized, rehydrated, and subjected to antigen retrieval in pH 6 Citrate buffer, at 96 °C for 30 min. After application of 0.4% Triton for 30 min, 3% H_2_O_2_ for 30 min and 10% Normal Goat Serum (Vector Laboratories, Newark, CA, USA) for 1 h, the sections were incubated overnight at 4 °C with a rabbit polyclonal antibody anti-RNF182 (Invitrogen; 1:100, Carlsbad, CA, USA). After washing in PBS, we applied a biotinylated anti-rabbit secondary antibody (1:100; Vector Laboratories) for 1 h, Streptavidin (1:100; Vector Laboratories, Newark, CA, USA) for 1 h and finally DAB (Sigma Aldrich, St. Louis, MO, USA). After counterstaining with Mayer’s hematoxylin (Diapath, Bergamo, IT, Italy), the sections were analyzed under a light microscope, at 100× magnification. In each section we performed a count of RNF182-positive cells compared to the total cells in 10 different fields. Statistical significance was determined by Unpaired *t*-test, considering a *p*-value * < 0.0001 statistically significant.

### 2.4. Protein Extraction and Western Blot Analysis

Proteins were extracted from frozen tissues of patients with Triton X-100 lysis buffer (10 mM Tris-HCl, 1 mM EDTA, 150 mM NaCl, 1% Triton X-100, 1 mM NaF, 1 mM Na_4_P_2_O_7_, 1 mM Na_3_VO_4_, and 1× protease inhibitors). After quantitation by Bradford Assay, 20 µg of total lysate were separated by sodium dodecyl sulfate-polyacrylamide gel electrophoresis (SDS-PAGE) and transferred onto Immuno-Blot^®^ PVDF Membrane. The membrane was incubated with 5% milk solution for 1 h at room temperature and then overnight at 4 °C with rabbit polyclonal anti-RNF182 (dilution 1:500, Invitrogen). The signals were detected by a digital scanner and quantified with Image Lab 6.1 Software (Bio-Rad Laboratories, Inc., Hercules, CA, USA). For protein normalization, the membrane was incubated with mouse monoclonal antibody anti-GAPDH (dilution 1:1000; Santa Cruz Biotechnology, Dallas, TX, USA). Data analysis and graphic representation were carried out by GraphPad Prism 8. Experiments were performed in quadruplicate and data were expressed as fold change. Statistical significance was determined by Unpaired *t*-test, considering a *p*-value * < 0.05 statistically significant.

### 2.5. RT-qPCR

Total RNA was extracted from a cohort of frozen tissues biopsies of patients by using the Direct-zol RNA MicroPrep (ZYMO RESEARCH, Irvine, CA, USA), while cDNA was synthesized using the SuperScript™ III First-Strand Synthesis System (Invitrogen). cDNA was then amplified by using the predesigned *rnf182* gene-specific primers (Forward 5′-TTGTGCCAAATGCCTCTACA-3′ and reverse 5′-GTGAGCAGCAGCTCAGTAGG-3′) with a sensitive Real-Time 2xHS-PCR SYBR Master Mix (A&A Biotechnology, Gdynia, Poland). All samples were run in triplicate on quantitative PCR plates and β-actin (Forward 5′-GCCAGACAGCACTGTGT-3′ and reverse 5′-GTGCGTGACATTAAGGAG-3′) was used as a reference gene. Data analysis and graphic representation were carried out by GraphPad Prism 8. The relative abundances of mRNA were subsequently analyzed using the 2(−ΔCt) method.

### 2.6. RNF182 Gene Silencing

Continuous GBM cell line U87MG was seeded with 4 × 105 cells in DMEM (Dulbecco’s Modified Eagle’s Medium) with 10% FBS (Fetal Bovine Serum) and without antibiotics. Cells were transiently transfected with RNF182 siRNA (sc-95222, Santa Cruz Biotechnology, Inc.) at a final concentration of 100 nM by using Lipofectamine 2000 (Invitrogen). U87MG CT were also transfected with a scrambled RNA, which does not target any gene, to simulate the same experimental condition and used as a negative control. The transfections have been verified by Western blot analysis using specific antibody anti-RNF182 (dilution 1:500, Invitrogen). As loading control an antibody anti-GAPDH (dilution 1:1000; Santa Cruz Biotechnology) was used. Data analysis and graphic representation were carried out by GraphPad Prism 8. Experiments were performed in triplicate and data were expressed as fold change. For Western blotting analysis, statistical significance was determined by Unpaired *t*-test, considering a *p*-value of ** <0.005 statistically significant.

### 2.7. Proliferation Assay

U87MG at 72 h of transfection with rnf182 siRNA were seeded in 48-multiwell plates, 1 × 10^4^ cells per well, in DMEM supplemented with 10% FBS (Fetal Bovine Serum), 2 mmol/L-glutamine, 100 IU/mL of Penicillin, and 100 g/mL of Streptomycin. Cell counts were performed at 24, 48, and 72 h using a Burker chamber. Graphs were plotted with GraphPad Prism 8. Statistical analysis of plotted curve was performed using 2-way ANOVA, multiple comparison **** *p* value < 0.0001. The images were acquired by EVOS FL Microscope at 10× magnification: scale bar 400 μm.

## 3. Results

### 3.1. In Silico Characterization of RNF182 in GBM

**Figure 1:**
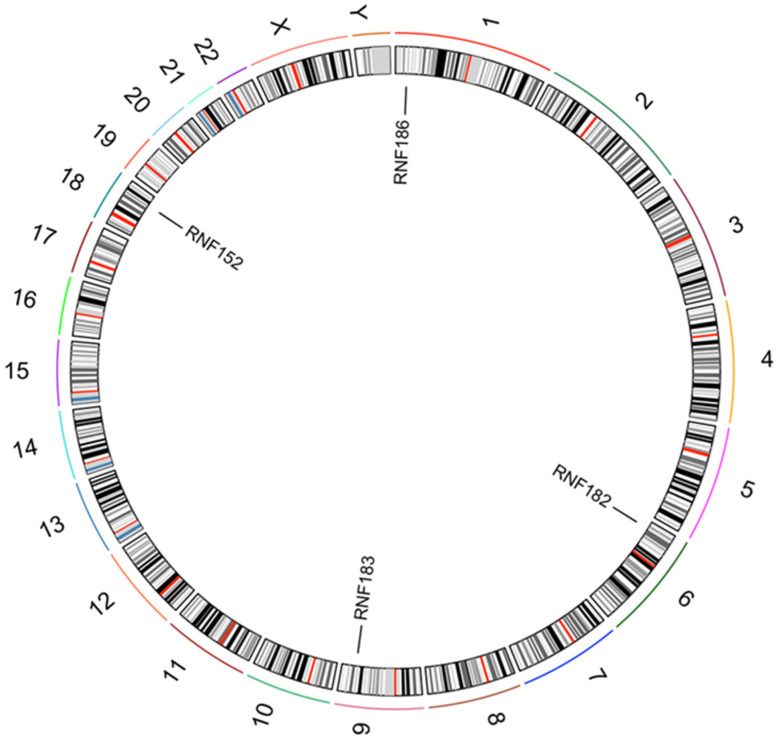
Location of RNF183 family members on chromosomes. RNF182 is represented on chromosome 6, RNF152 on chromosome 18, RNF183 on chromosome 9, and RNF186 on chromosome 1.

In this paper, we focus on RNF182, an E3 ligase predominantly expressed in the nervous system and belonging to the RNF183 family. This family is characterized by closely related genes encoding a RING-finger domain (C3HC4) at the N-terminus and one or two predicted transmembrane domains at the C-terminus, demonstrating high homology. Among the RNF183 family members (Figure 1), RNF182 is the least reported. Initially identified as a gene upregulated in the postmortem brains of patients with Alzheimer’s disease, RNF182 is also expressed in differentiated Ntera2 neurons and has been shown to be upregulated in response to oxygen and glucose deprivation [24]. However, its expression and role in glioblastoma have not yet been investigated.

**Figure 2:**
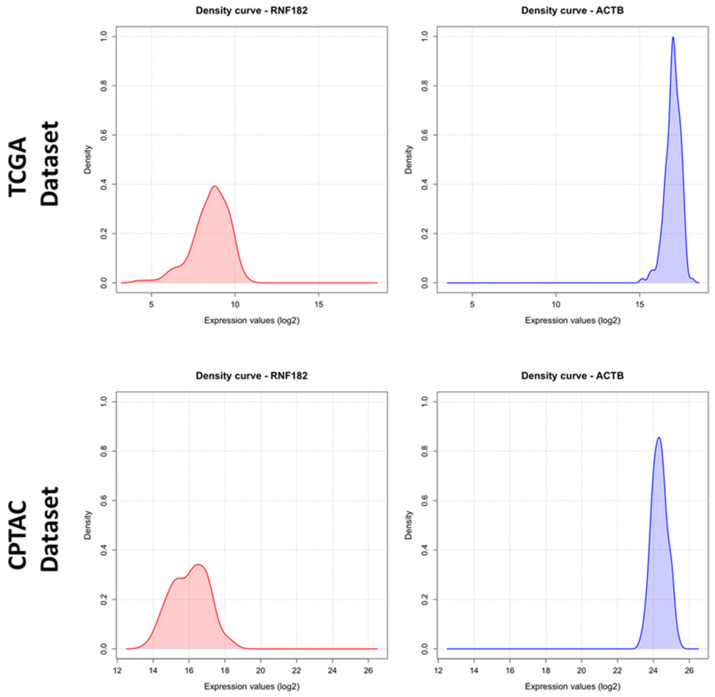
The graph displays a density curve representing the distribution of log_2_-transformed expression values of RNF182 and ACTB (housekeeping gene). The *x*-axis represents expression values, while the *y*-axis indicates the frequency of these values.

**Figure 3:**
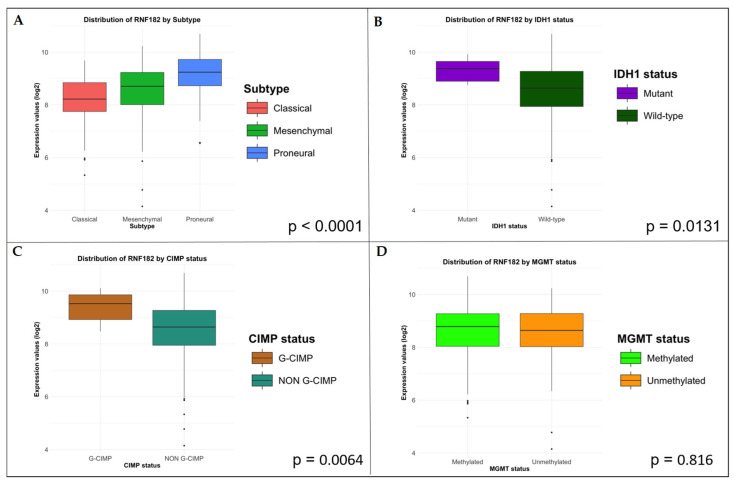
The image contains boxplots illustrating RNF182 expression under different conditions. The *x*-axis represents clinical variables, while the *y*-axis represents RNF182 expression levels. (**A**) The boxplot displays RNF182 expression across various clinical subtypes, showing that the Proneural subtype exhibits a statistically significant increase in RNF182 expression compared to other subtypes. (**B**) The IDH1 status boxplot indicates that samples with mutated IDH1 exhibit significantly higher RNF182 expression than those with wild-type IDH1. (**C**) The CIMP status boxplot shows that the G-CIMP phenotype is associated with a statistically significant increase in RNF182 expression compared to non-G-CIMP samples. (**D**) The MGMT promoter methylation status boxplot reveals that RNF182 expression is not significantly affected by MGMT promoter methylation status.

RNF182 exhibits a wide range of expression in GBM (Figure 2). Standard statistical analyses are summarized in Supplementary Figures S1 and S2. Statistical analysis revealed a significantly higher expression of RNF182 in the Proneural subtype (*p* < 0.0001), in tumors with IDH1 mutations (*p* = 0.0131), and in cases exhibiting the CpG island methylator phenotype (*p* = 0.0064) (Figure 3A–C). However, no significant differences were observed in the methylation status of the MGMT promoter (*p* = 0.816) (Figure 3D).

### 3.2. Biological Relevance of RNF182 in Gliomas

In order to understand the complex signaling networks of RNF182 in gliomas of different grades it was essential to quantify protein and gene expression and to examine the biological relevance of RNF182 in GBM. We analyzed 36 biopsy samples obtained from Neuromed patients following surgical resection of brain tumors, who provided informed consent for research studies. Of these 36 patients, 18 were affected by low-grade glioma and 18 were affected by GBM. The low-grade glioma group consisted of a heterogeneous adult population with a mean age of 50 years; most patients harbored an IDH1 mutation and 1p/19q co-deletion. The GBM group comprised a heterogeneous adult population with a mean age of approximately 60 years; among these, only four patients carried an IDH1 gene mutation (Table 1).

**Figure 4:**
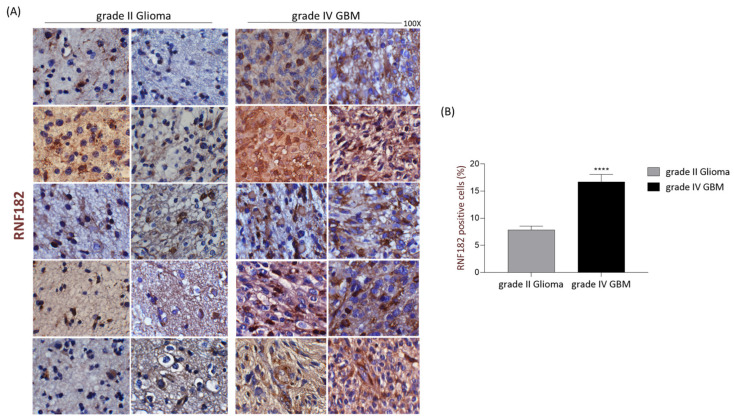
(**A**) Representative images of tissue sections biopsies from human grade II glioma and grade IV GBM immunostained with anti-RNF182 antibody (brown) and then analyzed by optical microscope. Images are acquired at 100× magnification; scale bar = 50 μm. (**B**) Graph represents the density (%) of RNF182-positive cells for 10 fields in low-grade (*n* = 18 samples) and high-grade gliomas (*n* = 18 samples) as described above; statistical significance considering *p* value: **** < 0.0001; Unpaired *t*-test.

**Figure 5:**
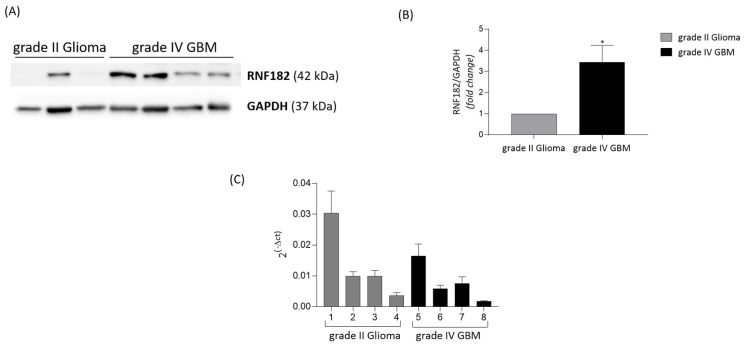
(**A**) Representative Western blot of RNF182 antibody on human glioma grade II and grade IV GBM. (**B**) Densitometric analysis (fold change) of RNF182 protein in human glioma grade II and grade IV GBM samples. GAPDH is used as loading control. Data are presented as fold chance of four independent experiments. Statistical significance: *p* value: * < 0.05. Unpaired *t*-test. (**C**) RT-qPCR of *rnf182* gene in low- and high-grade glioma samples. β-actin is used as control gene. The experiments are performed in triplicate for two individual experiments. The original Western blot figures can be found in File S1.

We initially examined the expression profile of RNF182 in 36 human glioma samples performing a comparative immunohistochemistry analysis on tissue slides with various grades of glioma. As shown in Figure 4A,B, the immunohistochemical analysis revealed a significant increment of RNF182 protein in high-grade compared to low-grade gliomas. In comparison to low-grade gliomas, GBM samples seem to have a greater percentage of RNF182-positive cells, exceeding 50%. The total proteins were extracted from frozen tissue biopsies and then analyzed by using Western blot analysis, in order to validate the immunohistochemistry results. Grade IV gliomas exhibited higher RNF182 protein expression than grade II gliomas, confirming outcomes reported in a previous investigation (Figure 5A,B). Therefore, we used reverse transcription-real-time quantitative polymerase chain reaction (RT-qPCR) to examine the expression of rnf182 in a cohort of tissues from grade II and grade IV gliomas. rnf182 RT-qPCR normalized with the control gene β-actin did not show significant expression changes in various grades of gliomas analyzed (Figure 5C).

### 3.3. RNF182-Mediated Regulation of GBM Cell Proliferation

**Figure 6:**
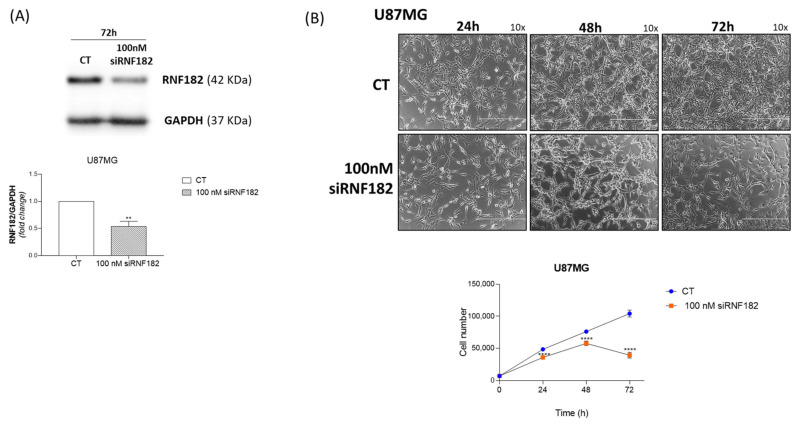
(**A**) Western blot analysis of RNF182 protein in U87MG transfected cells with 100 nM RNF182 siRNA. Densitometric analysis (fold change) of RNF182 protein in U87MG cells transfected with 100 nM RNF182 siRNA. GAPDH is used as loading control; statistical significance ** *p* value < 0.005. Unpaired *t* test. (**B**) Representative images and plotted growth curves of U87MG transfected cells with 100 nM of RNF182 siRNA or scrambled siRNA (CT). All experiments are conducted in triplicate (*n* = 3 biological replicates). The images are acquired by EVOS FL Microscope at 10× magnification: scale bar 400 μm. Statistical analysis of plotted curve is performed using 2-way ANOVA, multiple comparison **** *p* value < 0.0001. The original Western blot figures can be found in File S2.

To evaluate the putative involvement of RNF182 protein in GBM development and in growth regulation, we performed the silencing of the *rnf182* gene and evaluated cell proliferation. U87MG GBM continuous cell line was starved with complete DMEM 0.5% FBS for 48 h and subsequently transfected with duplex RNA RNF182 antisense for 72 h in order to evaluate the effect of the silencing on cell proliferation. Western blot examination clearly demonstrated the inhibitory effect of siRNA RNF182 on the expression of RNF182 protein. Treatment with 100 nM RNF182 siRNA reduced RNF182 protein expression at 72 h post-transfection (Figure 6A). In order to investigate the effects of siRNA RNF182 on GBM cell growth over time, cells were transfected with either siRNA scramble control or 100 nM siRNA RNF182. The cells were then harvested at different time points (24, 48, and 72 h) for cell proliferation assay and Western blot analysis at 72 h. The results showed that RNF182 siRNA significantly suppressed the proliferation of U87MG cells in a time-dependent manner. Compared with control cells, a significant growth-inhibitory effect was observed as early as 24 h post-transfection, with inhibition progressively increasing and reaching a maximum at 72 h after transfection (Figure 6B).

## 4. Discussion

Glioblastoma multiforme (GBM) is the most aggressive glioma, marked by invasive tumor cells, cellular heterogeneity, and glioma stem-like cells. Key dysregulated pathways include p53, RTK/RAS/PI3K, and RB [26,27,28]. Standard treatment combines surgery, radiotherapy, and temozolomide [13]. However, this treatment has limited effectiveness due to the tumor location and infiltration extent, and the potential brain damage from high doses of radiation. The TMZ effect, which blocks the cell cycle and activates apoptosis, is often hindered by DNA repair systems, which rely on O6-methylguanine methyltransferase (MGMT) [29]. Studies have been conducted to develop more effective therapies for GBM. New strategies, such as nanoparticles, immunotherapy, oncolytic viruses, or compounds with synergistic effects to TMZ, have been suggested to improve treatment efficiency. The chance of survival is significantly worse for older individuals with the disease [30,31]. Due to a lack of knowledge regarding the molecular pathways behind gliomagenesis, drug-based curative treatments are not the best option for treating gliomas. Furthermore, therapeutic delays for gliomas are caused by the imprecise and limited detection of early-stage gliomas gliomagenesis [32,33].

It has recently been established that genetic markers are useful clinical indicators that can be precisely assessed to forecast the course of gliomas [34]. Accordingly, optimizing current methodologies and introducing innovative techniques are imperative to improve the prognosis of patients with gliomas. The UPS-mediated proteolysis of cellular proteins is a complex mechanism regulating intricate biological interactions essential for cell survival, growth, and metabolism [35,36]. Consequently, the dysregulation of the UPS is causally linked to numerous human diseases, such as neurodegeneration and cancer [37]. Adjusting the total UPS function is a treatment approach currently undergoing evaluation for addressing various human conditions. Nonetheless, the use of it is restricted due to non-specific side effects and toxicity issues. A different strategy, focusing on the activity levels of particular E3 ligases, is appealing and potentially a more successful therapeutic approach [38,39]. Changes in the expression profile of components of the ubiquitin ligase complexes have been reported in diverse tumors [40,41]. Ubiquitin Conjugating Enzyme E2 S (UBE2S), an ub-conjugating enzyme that regulates cell cycle progression, is upregulated in high-grade astrocytoma [42]. The UBE2S was first recognized as an essential component in regulating substrate ubiquitination during the later stages of the complex’s functionality. In recent years, UBE2S has gained prominence as a vital epigenetic modification associated with various diseases, such as myocardial ischemia, viral hepatitis, and particularly, cancer. Increasing evidence indicates that UBE2S is instrumental in several human cancers, including breast cancer, lung cancer, and hepatocellular carcinoma, among others [43]. Nevertheless, a thorough review of UBE2S in the context of human tumor research is still lacking. Furthermore, the investigation into the role of UBE2S in developing resistance to cancer treatments is also addressed. The results imply that UBE2S presents potential as both a diagnostic and therapeutic target across multiple malignancies, thus paving the way for innovative cancer treatment strategies [44]. Somatic mutations of ubiquitin ligases have been identified in GBM and in other human malignancies, further supporting a pathogenic role of deregulated UPS in brain tumors [45]. Changes in E3 ubiquitin ligases, whether they are excessively produced or not produced enough, can cause cancer cells to become resistant to drugs. This happens because these changes affect important cell functions like fixing damaged DNA, programmed cell death, and how cells communicate with each other. For instance, when the E3 ligase UBR5 is produced in larger amounts, it is connected to resistance against platinum drugs in ovarian cancer [46]. On the other hand, when there is less of the ligase RNF125, it is linked to resistance against BRAF inhibitors in melanoma by influencing how cells signal. Alternatively, specifically stopping or changing these altered ligases could be a treatment approach to get around drug resistance [47]. Based on these findings, in our study, we investigated the role of a new ubiquitin ligase with unknown activity in glioblastoma. Our computational analysis suggested that RNF182 is upregulated in glioblastoma. Notably, RNF182 exhibits variable expression in GBM and is predominantly expressed in the proneural subtype, IDH1-mutated GBM and CpG island methylator phenotype. This in silico approach allowed us to assess whether RNF182 upregulation correlates with tumor grade and to assess its potential relevance to glioma progression. Motivated by these findings, we analyzed RNF182 expression in a cohort of 36 gliomas of different histopathological grades. Immunohistochemical analysis of tissue biopsies revealed that RNF182 expression progressively increases from low-grade astrocytomas to GBMs, indicating a strong association between RNF182 E3 ligase levels and tumor malignancy. The observed correlation implies that higher RNF182 expression may contribute to the advancement of glioma. Since RNF182 has been reported to regulate the ubiquitination and degradation of p65 (RELA), a key subunit of the nuclear factor kappa B (NF-κB) pathway that drives target gene transcription and promotes tumor progression [48], the distinct pattern of RNF182 protein expression observed in our study led us to hypothesize that this protein may play a pivotal role in the transition of gliomas from low-grade to high-grade forms. Consistently, Western blot analyses confirmed a significant increase in RNF182 protein abundance in grade IV GBM samples compared with grade II gliomas, reinforcing the notion that RNF182 upregulation is a molecular feature of high-grade tumors. Moreover, quantitative RT-qPCR analysis revealed no significant differences in RNF182 mRNA expression between low-grade and high-grade gliomas. This apparent discrepancy between unchanged transcript levels and increased protein abundance suggests that RNF182 regulation in gliomas occurs predominantly at the post-transcriptional level, potentially involving altered protein stability, translation efficiency, or degradation pathways associated with tumor progression. To elucidate the functional significance of RNF182 in gliomagenesis, its expression was suppressed in U87MG cells for 72 h using an antisense RNA duplex. Silencing of RNF182 resulted in a pronounced decrease in cell proliferation, with up to 50% inhibition of cell growth. Taken together, these findings provide functional evidence supporting a pivotal role for RNF182 in sustaining GBM cell growth and tumorigenic potential. Importantly, this evidence positions RNF182 as a promising molecular target and may inform the development of more effective, target-oriented therapeutic strategies for aggressive human GBM.

## 5. Conclusions

In conclusion, genetic or proteomic alterations affecting components of the ubiquitin–proteasome system (UPS), or their regulatory partners, can disrupt protein homeostasis, leading either to the pathological accumulation of oncoproteins or to the accelerated degradation of tumor suppressors. In this context, elucidating the functional interplay between RNF182, the ubiquitin–proteasome system (UPS), and glioma progression is particularly relevant for the development of selective, effective, and less toxic therapeutic strategies. Although classical proteasome inhibitors have demonstrated antitumor potential in glioma models, their clinical application is limited by systemic toxicity and lack of specificity. Consequently, alternative approaches such as selectively targeting UPS-associated proteins or exploiting emerging technologies like proteolysis-targeting chimeras (PROTACs) represent promising avenues to modulate proteasomal degradation pathways with greater precision and therapeutic benefit in gliomas.

## Figures and Tables

**Table 1:** Characteristics of grade II gliomas and grade IV GBM samples derived from Neuromed patients, according to the 2016 WHO classification.

CASE	GENDER	AGE	DIAGNOSIS	IDH1	LOH	MGMT
1	M	47	GLIOMA II° WHO (Diffuse astrocytoma II)	MUT	19q	MET
2	M	20	GLIOMA II° WHO (Diffuse astrocytoma II)	MUT	Normal	MET
3	F	47	GLIOMA II° WHO (Anaplastic Oligodendroglioma III)	MUT	1p-19q	MET
4	F	63	GLIOMA II° WHO (Astrocytoma II)	MUT	1p-19q	MET
5	M	35	GLIOMA II° WHO (Diffuse astrocytoma II)	MUT	Normal	MET
6	F	49	GLIOMA II° WHO (Astrocytoma II)	MUT	Normal	MET
7	M	67	GLIOMA II° WHO (Diffuse astrocytoma II)	MUT	Normal	MET
8	M	69	GLIOMA II° WHO (Oligodendroglioma II)	MUT	1p-19q	MET
9	F	43	GLIOMA II° WHO (Diffuse astrocytoma II)	MUT	Normal	MET
10	F	31	GLIOMA II° WHO (Oligoastrocytoma II)	MUT	1p-19q	MET
11	M	29	GLIOMA II° WHO (Oligoastrocytoma II)	WT	10q	UNMET
12	M	19	GLIOMA II° WHO (Diffuse astrocytoma II)	MUT	Normal	MET
13	M	67	GLIOMA II° WHO (Oligodendroglioma II)	MUT	1p-19q	MET
14	F	57	GLIOMA II° WHO (Oligodendroglioma II)	MUT	1p-19q	MET
15	M	30	GLIOMA II° WHO (Diffuse astrocytoma II)	MUT	10q	MET
16	F	41	GLIOMA II° WHO (Oligodendroglioma II)	MUT	1p-19q	MET
17	M	44	GLIOMA II° WHO (Diffuse glial tumor II)	MUT	1p-19q	MET
18	M	43	GLIOMA II° WHO (Oligoastrocytoma II)	MUT	1p-19q	MET
19	F	54	GBM	WT	-	UNMET
20	M	56	GBM	WT	-	MET
21	M	62	GBM	WT	-	UNMET
22	M	67	GBM	MUT	-	MET
23	F	35	GBM	WT	-	MET
24	M	73	GBM	MUT	-	UNMET
25	M	46	GBM	MUT	-	MET
26	F	51	GBM	WT	-	UNMET
27	F	67	GBM	MUT	-	UNMET
28	M	47	GBM	WT	-	MET
29	M	81	GBM	WT	-	UNMET
30	F	80	GBM	WT	-	MET
31	M	52	GBM	WT	-	UNMET
32	F	77	GBM	WT	-	UNMET
33	F	55	GBM	WT	-	UNMET
34	M	70	GBM	WT	-	UNMET
35	M	66	GBM	WT	-	UNMET
36	M	70	GBM	WT	-	UNMET

## Data Availability

The data that support the findings of this study are available from the corresponding author upon reasonable request.

## Supplementary Materials

The following supporting information can be downloaded at: https://www.mdpi.com/article/10.3390/cancers18081217/s1, Files S1 and S2: The original Western blot figures; Supplementary Figures S1 and S2: Standard statistical analysis.

## Abbreviations

GBM	Glioblastoma
IDH1	Isocitrate dehydrogenase 1
TERT	Telomerase reverse transcriptase
CDKN2A	Cyclin-dependent kinase inhibitor 2°
G-CIMP	Glioma CpG island methylator phenotype
TMZ	Temozolomide
MGMT	O6-methylguanine-DNA-methyltransferase
UPS	Ubiquitin–proteasome system
ATP6V0C	ATPase H+ transporting V0 subunit c
RNF182	Ring finger protein 182
TLR	Tool-like receptors
NF-κB	Nuclear Factor kappa-light-chain-enhancer of activated B cells
RELA	v-rel avian reticuloendotheliosis viral oncogene homolog A
AD	Alzheimer’s disease
WGCNA	Weighted gene co-expression network analysis
GO	Gene ontology
DAVID	Visualization and integrated discovery
U87MG	Uppsala 87 malignant glioma
PBS	Phosphate-buffered saline
DAB	3,3′-diaminobenzidina
SDS-PAGE	Sodium dodecyl sulfate-polyacrylamide gel electrophoresis
DMEM	Dulbecco’s modified Eagle’s medium
FBS	Fetal bovine serum
RTK/RAS/PI3K	Tyrosine kinase receptor/Rat sarcoma virus/phosphoinositide 3-kinase
EDTA	Ethylenediaminetetraacetic acid
GAPDH	Glyceraldehyde-3-phosphate dehydrogenase
siRNA	Small interfering RNA
SOX2	Sex-determining region Y-box 2
MUT	Mutate
WT	Wild-type
WHO	World Health Organization
GSCs	Glioma stem-like cells
RT-qPCR	Reverse transcription-quantitative polymerase chain reaction
UBE2S	Ubiquitin conjugating enzyme E2 S
UBR5	Ubiquitin protein ligase E3 component N-recognin 5
RNF125	Ring finger protein 125
PROTACs	Proteolysis targeting chimeras
