# Supplementary figures and images for: Overexpression of the Ubiquitin Ligase RNF182 Is Associated with High-Grade Gliomas
Source: Cancers (Basel). 2026 Apr 11;18(8):1217. doi: 10.3390/cancers18081217 (PMC13114817; doi:10.3390/cancers18081217)

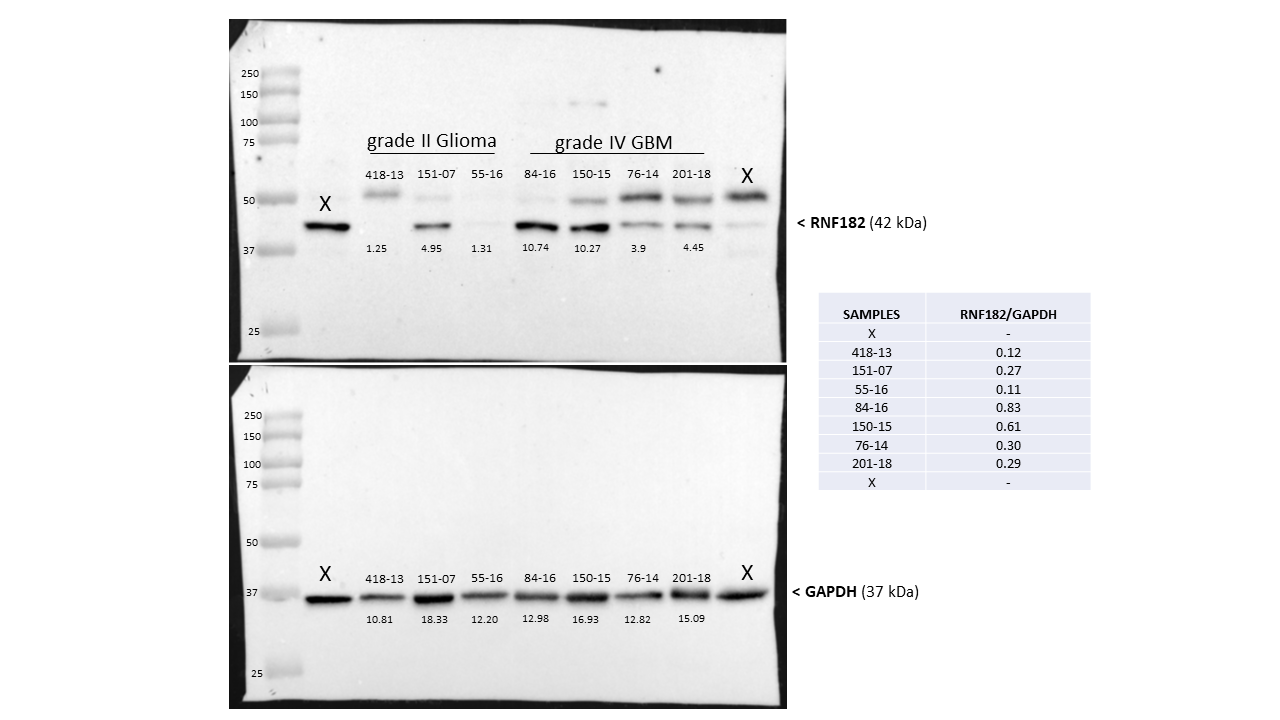

Supplement: Supplementary file 1 [file cancers-18-01217-s001.zip › File S1.tif]

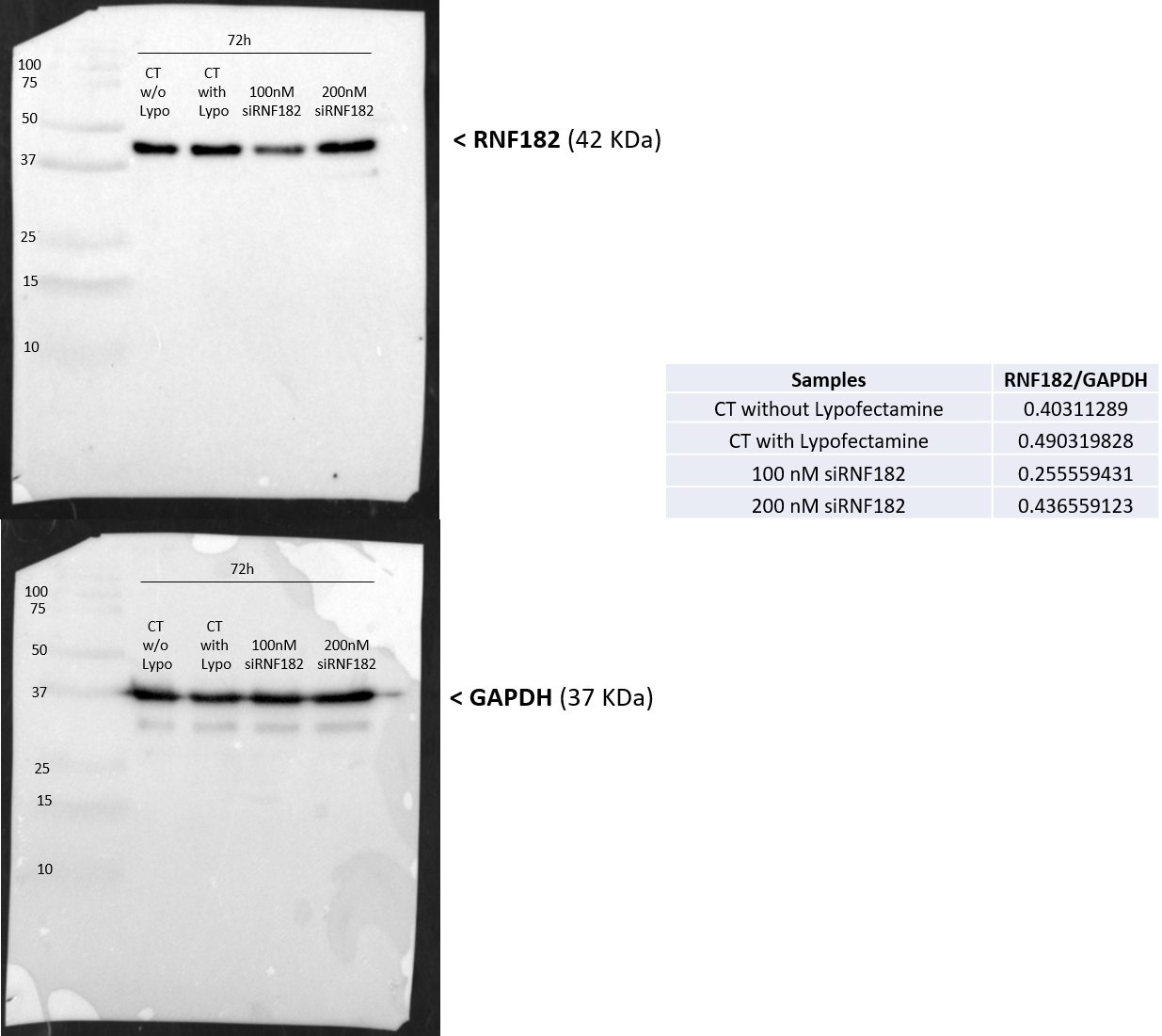

Supplement: Supplementary file 1 [file cancers-18-01217-s001.zip › File S2.tif]

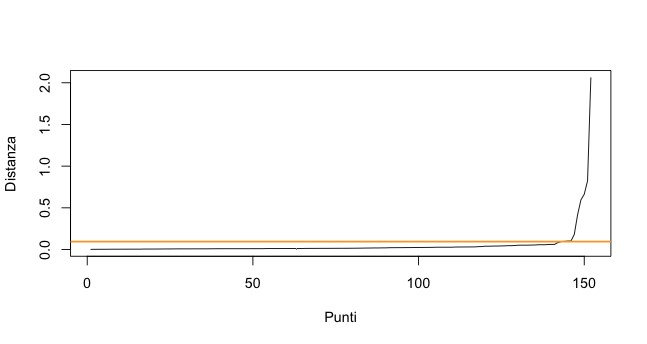

Supplement: Supplementary file 1 [file cancers-18-01217-s001.zip › Supplementary Figure S1.tif]

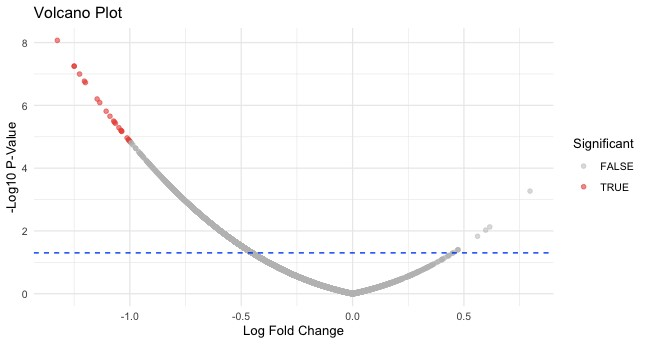

Supplement: Supplementary file 1 [file cancers-18-01217-s001.zip › Supplementary Figure S2.tif]
